# Impact of COVID-19 on an established physical activity and behaviour change support programme for cancer survivors: An exploratory survey of the Macmillan Move More service for Northern Ireland

**DOI:** 10.1007/s00520-021-06165-1

**Published:** 2021-04-03

**Authors:** Malcolm Brown, Dominic O’Connor, Claire Murphy, Maura McClean, Alexandra McMeekin, Gillian Prue

**Affiliations:** 1 School of Nursing and Midwifery, Queen’s University Belfast, Medical Biology Centre, 97 Lisburn Road, Belfast, BT9 7BL Northern Ireland; 2 Macmillan Cancer Support, Belfast, Northern Ireland

**Keywords:** COVID-19, Cancer, Macmillan Move More NI, Physical activity, Service evaluation

## Abstract

**Purpose:**

The recent coronavirus pandemic (COVID-19) has affected the delivery of routine cancer care and supportive services. The Macmillan Move More Northern Ireland (MMNI) programme provides access to physical activity and behavioural change support before, during and after cancer treatment. This evaluation details the impact of the pandemic on the MMNI participants and identifies methods to adapt service delivery.

**Methods:**

A multiple-choice and short answer online survey was sent to 730 MMNI participants, to investigate the impact of the initial, national COVID-19 lockdown. Specifically, the survey examined physical activity patterns, the physical/emotional/social impact of restrictions and attitudes towards digitally supported exercise. Free text responses were analysed thematically with findings verified within the research team.

**Results:**

377 participants completed the survey (52% response rate). 50% of respondents had breast cancer, with 36 other diagnoses registered (82% were female). Participants reported physical activity levels decreased during restrictions, citing isolation; declining health/fitness; lack of access and motivation. The dataset trended towards women and those diagnosed with breast cancer. 71% reported the pandemic impacted their physical (n=119) and/or psychosocial (n=231) wellbeing. Many respondents were availing of digitally supported exercise, whilst half of males did not engage (46%). Finally, 80% of respondents were interested in using a MMNI smart application.

**Conclusion:**

The COVID-19 pandemic has affected participant physical activity levels. Supervised classes were the most popular (pre-pandemic), with enforced leisure centre closures prompting this reduction. The pandemic has negatively affected the psychosocial wellbeing (mental health) of participants, compounded by the restrictions imposed on the traditional delivery of MMNI. This impact is felt equally across cancer types. Participants with breast cancer are the most engaged in using digital technology to access exercise. Although underrepresented, men require greater targeting to ensure equality in access to online services.

**Supplementary Information:**

The online version contains supplementary material available at 10.1007/s00520-021-06165-1.

## Introduction

Severe acute respiratory syndrome coronavirus 2 (SARS-CoV-2) is the viral strain responsible for the novel coronavirus disease 2019 (COVID-19) pandemic. Since its emergence in December 2019, the viral outbreak has subsequently evolved into a global public health emergency. COVID-19 is associated with lethal respiratory infections in humans and affects people of all ages; however, older adults and those with comorbid medical conditions are at greater risk [1]. The virus is primarily spread through direct (person-to-person) or indirect (surfaces) contact transmission via respiratory secretions [2]. As of 21^st^ February 2021, there are 4.1 million confirmed cases in the UK (110,979 in Northern Ireland) with ~ 120,000 deaths [3]. The UK government responded to the surge in cases by implementing widespread transmission prevention measures. Without an effective vaccination programme, physical distancing alongside good personal hygiene (hand hygiene, face coverings etc) appear to be the most effective measures to control transmission [4, 5]. The UK entered a national lockdown in March 2020 and in recent months restrictions have been relaxed and reimposed. However, mitigation measures have severely impacted the provision of clinical services including routine cancer care and supportive services [6]. In fact, the extent of the pandemic has created a backlog of undiagnosed cases, that even additional resources will take significant time to rectify [7].

Each year, over 360,000 (>9,400 in Northern Ireland) new cancer diagnoses are reported in the UK, with numbers estimated to increase by 2% by 2035 [8]. With increasing numbers of cancer survivors, effective supportive care interventions are required. Accumulating evidence recognises regular exercise as an effective supportive care intervention which can induce many physiological and psychosocial benefits. These include improved tolerance to cancer treatment-related toxicities, improved disease outcomes and better quality of life throughout survivorship [9]. Exercise training has been shown to improve cardiorespiratory fitness, muscular strength, body composition, fatigue and overall quality of life in individuals with cancer, with supervised exercise preferred to self-directed exercise [9, 10]. However, the restrictions imposed in response to the COVID-19 pandemic prevents the delivery of face-to-face exercise sessions. In response, exercise providers have been forced to innovate using digital technology to provide access [6], whilst service users have embraced these innovations to remain active.

Macmillan Move More NI (MMNI) is a physical activity referral programme in partnership with Macmillan Cancer Support and the eleven district councils across Northern Ireland, each containing a MMNI coordinator. As qualified exercise specialists, coordinators are responsible for implementing the Macmillan Physical Activity Behaviour Change Care Pathway. The overarching aim of the programme is to ‘*ensure that everyone living with cancer in Northern Ireland is aware of the benefits of physical activity and is enabled to choose to become and stay active, at a level that is right for them*’. This is achieved by providing a personalised plan of supervised physical activity and exercise, information and support to empower behavioural change. The programme aims to help people become and remain active, improve their general fitness, assist in managing cancer treatment, and improve their quality of life, ultimately enjoying the many renowned benefits of exercise [9]. Traditionally this service is delivered face-to-face at local leisure centres throughout NI, by specialist coordinators that tailor exercise prescription. As a result of COVID-19 restrictions, the MMNI service has adapted (remotely using digital technologies e.g. Zoom, YouTube etc) to enable continued delivery. Whilst these changes have been imposed by the COVID-19 pandemic, it underscores the need for scalable remote interventions in the longer term [11]. Innovative technological solutions are cost-effective and have the potential to broaden accessibility. Providing a dual service of face-to-face classes (once restrictions are lifted) and remote access, may help improve overall service delivery.

However, the impact of COVID-19 on the MMNI programme and the effects of modified service delivery on its users is unknown. Consequently, a service evaluation was conducted, detailing the impact of the COVID-19 restrictions on participants. As COVID-19 restrictions are currently implemented globally, the results of this survey may help guide service innovation for similar community-based programmes. As detailed above, learning from the transition to remote service delivery and participant experiences, during a global pandemic or similar public health crisis, could be useful in designing and delivering a person-centred service (using mixed delivery methods) to improve access for all.

## Methodology

### Design and Participants

A cross-sectional sample of participants were actively recruited over a 3-week period (8^th^ to 28^th^ June 2020). Participants were eligible for inclusion if they (1) received a cancer diagnosis and (2) participated in the MMNI programme. All eligible participants provided informed consent online, prior to accessing the survey. Consent was sought after participants reviewed the survey purpose and privacy statement. All participants were at least 18 years of age, with responses stored in accordance with the Data Protection Act (2018). Prior to commencing, we sought advice from research governance pertaining ethical review. As a service evaluation, it does not fall under the usual ethical procedures and instead we completed a Data Privacy Impact Assessment (DPIA), under the direction of the University Information Compliance Unit. The research team at Queen’s University Belfast were blinded to patient identity and all data collection, maintaining anonymity throughout.

### Procedures

Participants were identified by MMNI via their user database. Eligible participants received an anonymous multiple-choice questionnaire and short answer online survey, investigating the impact of COVID-19 on the MMNI service. The questionnaire and short answer survey was developed by researchers at Queen’s University Belfast, using Microsoft Forms and refined through consultation with MMNI, prior to a piloted release. Upon review of the pilot data and satisfactory feedback, the questionnaire and short answer survey was distributed to participants via the MMNI coordinators. Participants received a hyperlink to access the online survey. Data was collected and stored electronically for the duration of the recruitment period. Following distribution, participants received weekly reminders by email, WhatsApp, text or telephone to ensure maximum uptake. Each participant completed the survey once. For inclusiveness, if participants were unable to access the survey directly a Move More coordinator had the capacity to complete on the participants behalf, through telephone consultations.

The online survey was provided in English and consisted of 21 questions (5 open-ended). Participants were required to provide a response for each question. Limited, non-identifiable demographic data was collected (i.e. gender; cancer diagnosis). Participants were asked to report the frequency and type of exercise (i.e. closed questions) completed prior to and during COVID-19 restrictions. Two open-ended short answer items provided participants the opportunity to describe the impact of COVID-19 restrictions on their life and any concerns regarding a return to MMNI services when restrictions are eased. The various impacts reported were coded and grouped within two main categories: (1) Psychosocial, and (2) Physical. The emphasis of the remainder of the survey focused on digitally supported exercise. Participants were asked to simply report if they presently used digital technology to access exercise (i.e. yes / no) and if they might consider using digital platforms (e.g. Zoom, FaceTime, mobile apps) for access in future (i.e. yes / no). Finally, participants were asked to rate on a Likert scale (not at all - very much) the influence of common barriers to exercise participation during COVID-19 restrictions. Participants received no incentive to participate.

### Data analysis

Demographic information was summarised using descriptive statistics. Participant responses obtained from the Likert scale were also summarised descriptively and average scores were calculated. The full free text response data set was analysed thematically according to Miles and Huberman’ [12] techniques of labelling, coding, categorising and theme development. The process involved identifying commonalities in the data set and searching and comparing the free text responses to identify relationships and themes. Constant comparative techniques were used to ensure all perspectives were represented in the analysis, and deviant cases examined. Findings were verified and discussed by the research team at each stage to assess accuracy and credibility of the interpretation, promote inter-rater reliability and ensure rigour [13]. Some non-identifiable, verbatim comments are included in the supplementary material.

## Results

### Participants

In total, 377 (52% response rate) MMNI service users responded to the survey (325 [86%] completed by participants; 52 [14%] completed by MMNI coordinators on behalf of participants). Participant demographics can be observed in Table 1. Participants were predominantly female (n=309, 82%), with breast cancer reported as the most prevalent primary cancer site (n=190). Thirty-six additional cancer sites were reported by 4 participants or fewer (not reported in Table 1).

**Table 1:** Participant demographics

Parameters	No. of participants (%)
Gender	
Female	309 (82)
Male	68 (18)
Tumour site	
Breast	190 (50.3)
Prostate	28 (7.4)
Colorectal	22 (5.8)
Lymphoma	18 (4.8)
Ovarian	14 (3.7)
Lung	10 (2.7)
Endometrial	9 (2.4)
Uterine	8 (2.1)
Thyroid	7 (1.9)
Kidney	6 (1.6)
Oesophageal	5 (1.3)

### Exercise frequency

Exercise frequency before COVID-19 restrictions and during COVID-19 restrictions are reported in Table 2. The percentage of participants who were not regularly active increased from 4% prior to COVID-19 restrictions to 21% during COVID-19 restrictions. The number of participants reporting physical activity levels of 1–2, 3–4, and 5–6 days / week decreased by 4, 9 and 5 % respectively during COVID-19 restrictions. The number of participants reporting daily physical activity increased by 1% during restrictions.

**Table 2:** Physical activity frequency prior to and during COVID-19 restrictions

	Physical activity level prior to COVID-19 restrictions		Physical activity level during COVID-19 restrictions	
	No. of participants	%	No. of participants	%
Not regularly active	15	4	77	21
1 – 2 days / week	99	27	87	23
3 – 4 days / week	137	36	101	27
5 – 6 days / week	65	17	47	12
Everyday	61	16	64	17

### Exercise location

Prior to restrictions the primary setting for exercise was MMNI classes in a leisure facility (n=344, 91%). During restrictions, indoor (n=216) and outdoor (n=217, 57%) home-based exercise were most common. Forty-four (12%) participants reported no exercise during restrictions.

### Type of physical activity / exercise under COVID-19 restrictions

During COVID-19 restrictions participants reported walking (n=283, 75%) as the most common form of physical activity completed. Other popular forms of physical activity included gardening (n=149, 39%), online MMNI classes (n=137, 36%) and their own form of home-based activity, not provided by MMNI (n=159, 42%).

### Impact of COVID-19 restrictions

Most participants (n=268, 71%) felt the pandemic did have an impact on them, whilst 45 (12%) reported no impact, and 64 (17%) participants were not explicit in their response.

### Psychosocial impact

Most participants highlighted the psychosocial impact of COVID-19 restrictions (n=231, 61%). Psychosocial themes included 1) lack of social support / loneliness, 2) decreased motivation to exercise 3) fear, and 4) anxiety. Lack of social support / loneliness (n=185, 80%) was the most commonly reported psychosocial issues during restrictions (Table 3).

**Table 3:** Physical and psychosocial impacts of COVID-19 restrictions

Impact	
Psychosocial impact	Loneliness
	Lack of social support
	Decreased motivation to exercise
	Fear
	Anxiety / Depression
Physical impact	Fitness deterioration
	Health deterioration
	Increased pain
	Increased body weight
	Dietary changes

### Physical impact

The physical impact of COVID-19 restrictions was reported by 32% (n=119) of the participants. Physical themes included 1) deterioration in fitness, 2) deterioration in health, 3) increased pain, 4) increased body weight, and 5) changes in dietary habits.

### Returning to Move More services

Most participants (n=231, 61%) reported positive feelings about returning to MMNI services when restrictions permit, with no concerns raised (provided COVID-19 mitigation measures are strictly adhered). In contrast, 36% (n=134) of participants reported concerns of safety, social distancing and hygiene.

### Using digital technology to access physical activity / exercise

Most participants (n=233, 62%) were currently using digital technology to access exercise (Table 4). The most common device used was a smart phone (n=71, 30%), whilst Zoom (n=60, 26%) and YouTube (n=50, 21%) were the most common applications used. Of the 144 not currently using digital technology, 62 (43%) highlighted they were interested in using digital technology in future.

**Table 4:** Digital techologies utilised during COVID-19 restrictions

Digital technology	No of participants
Device	
Smart phone	71
PC / laptop	28
Tablet (i.e. iPad)	52
Smart watch	9
Smart television	11
Fitbit	24
Application / software	
Zoom	60
Facebook	9
YouTube	50
WhatsApp	20
Mobile apps (e.g. Strava)	14
Microsoft Teams	1
Internet	26
DVD	1

### Preferred method of Move More class delivery under COVID-19 restrictions

Most participants (n=183, 49%) stated they would prefer to access MMNI classes digitally via the MMNI YouTube channel, which has a library of pre-recorded exercise sessions. Move More exercise sessions delivered in real-time via platforms including Zoom and Microsoft Teams and through smartphone apps were also preferences amongst 155 (41%) and 108 (29%) participants, respectively.

### Barriers to exercise during COVID-19 restrictions

Participants (n=194, 52%) reported that they did experience difficulties engaging in exercise during COVID-19 restrictions, whilst 182 participants (48%) reported no difficulty. Isolation was reported as the greatest barrier to exercise (n=52, 27%), followed by declining health/fitness (n=44, 23%); lack of access (n=40, 21%) and lack of motivation (n=38, 20%).

### Evaluation of the Macmillan response to COVID-19 restrictions

There was widespread recognition and satisfaction that the MMNI programme were providing options to engage in physical activity / exercise, with most participants (n=263, 70%) happy with the proactive response to initial and ongoing restrictions. Of the 86 participants (23%) who felt more could be done to assist participation, common themes included outdoor exercise classes, greater flexibility with ‘live’ (i.e. Zoom) exercise classes, and technological support.

### Development of a MMNI App

The majority of participants (n=300, 80%) reported that they would be interested in using a MMNI smartphone App to receive or participate in live exercise. When asked how they would feel about using an App, the majority of participants (n=271, 72%) were positive. Common positive themes revolved around facilitating group contact and providing flexibility around current lifestyle. Although some individuals (n=61, 16%) did express negative emotions. Emerging negative themes focused on lack of technological proficiency and a preference towards face-to-face classes*.*

## Discussion

The aim of this service evaluation was to detail the impact of COVID-19 restrictions on people living with cancer, who engage in the MMNI programme and to evaluate perceptions on alternative approaches to service delivery. The societal restrictions imposed in response to the COVID-19 pandemic have limited the ability of exercise providers to deliver supervised, face-to-face exercise. This may have significant effects on wellbeing, since frequent exercise is recommended to help people living with cancer maintain both physical and psychosocial wellbeing [9]. The current study demonstrates the impact of COVID-19 restrictions on the physical and psychosocial wellbeing of MMNI service users, which has been further compounded by the restrictions imposed on MMNI capabilities to deliver face-to-face exercise classes. To continue MMNI services and promote exercise during restrictions, the Move More coordinators responded with service adaptations involving ‘live’ exercise sessions, facilitated through digital technology (e.g. Zoom), and the development of recorded exercise sessions uploaded to YouTube. This response was welcomed with a positive, large-scale adoption of digital technology for exercise provision, and further interest in digital based solutions.

Although interest and engagement in home-based exercise surged briefly when restrictions were originally implemented [14], disruptions to cancer exercise services such as MMNI removed an important supervised and supportive environment for cancer survivors to participate in group exercise. Consequently, cancer patients risk regressing to a sedentary lifestyle which may have a negative impact on their physical and psychosocial wellbeing [15]. Results from this evaluation confirmed the impact of reduced face-to-face MMNI services and subsequent reductions in exercise frequency. In agreement, a recent international, online survey of 1047 participants, reported a decrease in physical activity intensity levels and subsequent increase in daily sitting time, as a consequence of COVID-19 restrictions [16]. Similarly, a recent community-based physical activity programme for 61 cancer survivors reported reduced physical activity (67%) since the start of the pandemic, alongside impacts to mental health [17]. Interestingly, some respondents (i.e. the most active) managed to maintain high levels of activity, during COVID-19 restrictions, perhaps related to their levels of intrinsic motivation, self-confidence and psychosocial support, all of which are important components of exercise adherence [18].

Nonetheless, most respondents reported both physical and psychosocial impacts of restrictions including loneliness, loss of social support, loss of motivation, deterioration in fitness/health, and negative changes in body composition. This is a consistent finding, as a recent online survey of 1491 adults (inclusive of participants with chronic illness) reported decreased physical activity (48.9%) since the start of the pandemic and greater levels psychological distress (e.g. anxiety and depression) [19]. Even though breast cancer was heavily represented in the present survey, possibly a result of a finely tuned referral pathway to the service or the strength of the evidence base for exercise in breast cancer survival - lending to greater engagement, these themes were typical across gender and all cancer diagnoses. However, with the enforced loss of such essential community cancer exercise services during restrictions and the physical and psychosocial impact on wellbeing, this highlights the need for the rapid development of alternative interventions which can safely, and reliably deliver tailored exercise in participants homes whilst providing a social network of support [15]. While the COVID-19 pandemic appears to have impacted our sample population, we note that this may not be the case in wider sections of society. A recent lifestyle survey of 3533 respondents, investigated the immediate impact of the pandemic and recorded a slight increase in self-reported physical activity [20]. However, notable methodological differences exist within the sampling groups particularly age, health status, geography and potential subjective bias in self-reporting lifestyle behaviours.

Data from pre-COVID-19 studies in prostate cancer suggests that switching from supervised to home-based exercise may not confer any additional benefits to physical and psychosocial outcomes including fatigue, quality of life, and body composition [21–23]. Detrimental changes in body fat may also occur during a COVID-19 imposed lockdown [24] and may negatively impact metabolic health, and disease prognosis, highlighting the necessity for continued exercise support. Optimising an exercise stimulus and facilitating the intervention through digital technology, may help increase contact with participants and maintain motivation and adherence [15, 25]. The new age of COVID-19 restrictions means cancer exercise services will be required to adapt quickly to the changing environment. MMNI responded to the COVID-19 challenge with innovative service provision. This included a bank of pre-recorded exercise sessions uploaded to YouTube. Sessions created and uploaded to internet servers offer convenient access to unsupervised exercise for those with regular exercise habits, but may be unlikely to promote adherence in people affected by cancer [15]. Facilitating exercise with digital technology and offering supervised group-based exercise ‘live’ via digital platforms (e.g. Zoom), can introduce remote supervision, camaraderie and peer support, maximising adherence and effectiveness [26]. The success of these service changes is reflected in their widescale adoption. The results from this survey highlight a positive adoption of new exercise behaviours, with most participants accessing technology facilitated exercise, through Zoom. This is likely influenced by the ubiquity of digital / mobile technology for health, and its convenience and flexibility [27]. We anticipate that an integrated, purpose-built app incorporating the full repertoire of MMNI services (e.g. virtual / ‘live’ supervised exercise; uploaded exercise videos; behavioural support etc), provides a singular location to support participants, enhancing continuity in service delivery and engagement. Telehealth is thus emerging as a viable option for exercise delivery, during the pandemic [28, 29] and in the recent past has proved successful in improving physical and cognitive functioning, pain, strength, fatigue and global health status in cancer populations [30].

Although largely positive data supported the response of MMNI during restrictions, some concerns were raised regarding a lack of technological support. The need for more technological support is unsurprising given that most cancer survivors are over 65 years old [31], and despite the growing ubiquity of mobile technology in this age group, many may not have the technological proficiency to effectively use the technology to support exercise [32]. Supplementary technology-specific support (e.g. access and navigating digital platforms) is required to enable participants to fully utilise novel technologies, to facilitate their remote exercise programming. Indeed, the current data highlights an eagerness within the sample to adopt new technologies with the majority (n=300) keenly backing the development of a MMNI mobile app. This overwhelmingly positive response may have stemmed from their experiences of using digital technologies to support their exercise regime during restrictions (62%) or indeed the continued need to avail of support in general, using the medium of technology as an outlet. Similarly, a parallel survey conducted during the pandemic, explored alternative strategies for the delivery of exercise and social support, with participants expressing a preference to using digital technology for exercise programming and social support [17]. Since a lack of instruction and guidance is a common barrier in older adults [33], appropriate education and support is necessary. In addition, the involvement of all stakeholders in a user-centred design approach would likely optimise the App. Such an approach would ensure the App is evidence-based, theoretically informed and practical, including all the information and features to enable early uptake and implementation [34]. Given people with cancer may live with numerous side effects of treatment (e.g. fatigue; neuropathy; immunosuppression etc), inhibiting their commute to classes, as well as the present restrictions imposed, investment in a smart application may prove advantageous to ensure remote accessibility and engagement. The current circumstances (i.e. COVID-19 restrictions) have forced users to become innovative and embrace technology, so this may prove an optimal time to develop app-based delivery, whilst participants are receptive to change. For as long as restrictions persist, it could provide a pragmatic, cost-effective alternative for exercise delivery.

## Limitations

This service evaluation is not without limitations. The inclusion of free text questions within the survey aims to provide a deeper understanding of the participant experiences, however these should be interpreted with caution. Free text responses were often limited to one or two sentences which may reduce the potential to understand the context of the participant experience. In addition, a small number of participants did not provide free text responses, perhaps indicative of participant burden or indeed an uneasiness documenting negative experiences. We are reasonably confident the latter was not the case, given the reporting of both positive and negative experiences. A further and important limitation is the representation of the sample. Females with a diagnosis of breast cancer comprise half the respondents, with males underrepresented (18%). This suggests, that while common themes were reported for both genders, the results are more representative of female experiences. Equally, we cannot be sure that participants submitted a single response to the survey, without collecting enhanced identifiable information (e.g. name; date of birth; IP addresses). However, we mitigated the possibility of duplicate responses by providing strict instruction during the consenting process (to complete once) as well as a submission message at the end, so participants were in no doubt that their response was registered. Lastly, the self-reported nature of physical activity levels, might also present a limitation (e.g. reporting bias). Despite these limitations, this study demonstrates the impact of COVID-19 restrictions on MMNI participants, and the response of this service to national adversity.

## Conclusions and Recommendations

Findings confirmed reduced exercise participation amongst MMNI participants when face-to-face sessions ceased, in response to restrictions imposed. Although, the adoption of digital technology to help access exercise remotely was reported, participants described the negative impact of the restrictions on their physical and psychosocial wellbeing. Alternative methods of delivery introduced by MMNI to continue service provision were positively welcomed, creating further interest in developing tailored digital solutions to deliver targeted exercise. Such technology used alongside face-to-face sessions (when restrictions permit) has the potential to reach a larger population, including those who report competing interests and programme location as barriers [35] and would bring added benefit to those already enrolled. Below, we outline recommendations for future practice in the changing COVID-19 environment, based on the findings.

1. Whilst remote, digital support was readily received by participants, many are not technically proficient or have access to such technologies, given the majority of people living with cancer are over 65 years old [31]. Therefore, providing additional resource and/or support (e.g. written guidance by post) to ensure participants receive basic educational materials and practice on using digital applications is required.
2. Participants reported an eagerness to adopt digital technology. As such, the development of a mobile app appears a feasible option in this cohort, though stakeholder input is necessary.
3. Loneliness, isolation, and loss of social support are prominent issues. Therefore, a buddy system or MMNI champion should be explored, to enable the further provision of peer support.
4. A number of responses stated a willingness to partake in physical activity sessions outdoors, whilst indoor activities are prohibited. For as long as restrictions persist and where guidelines permit, a suitable outdoor MMNI programme should be considered.

Adopting mixed methods of exercise delivery is becoming essential when seeking to maximise the reach and engagement of people living with cancer, thus supporting exercise and behavioural change. Given the rapid response and subsequent versatility of the MMNI programme, it could be considered an example of best practice or model to follow for similar cancer support services.

## Supplementary Information

ESM 1 (DOCX 17 kb).

## Data Availability

Not applicable.
