# Supplementary material for: Impact of COVID-19 on an established physical activity and behaviour change support programme for cancer survivors: An exploratory survey of the Macmillan Move More service for Northern Ireland
Source: Support Care Cancer. 2021 Apr 3;29(10):6135–43. doi: 10.1007/s00520-021-06165-1 (PMC8019085; doi:10.1007/s00520-021-06165-1)
Supplement: Supplementary file 1 — (DOCX 17 kb). [file 520_2021_6165_MOESM1_ESM.docx]

Re: Evaluating the Impact of COVID-19 on the Macmillan Move More Service for Northern Ireland: Results from an Exploratory Survey.

Supplementary materials

Please find included below, under each of the subheadings, extracts from the free text responses to the survey.

*Psychosocial impact*

*“Whilst I have been lucky enough to get out for a walk each day and keep physically active, I have not been able to meet or interact with family and friends and I feel this has impacted on my mental wellbeing” (female, breast cancer).*

*“Missing our regular move more class, missing the social interaction with the rest of the group and our coordinator” (male, prostate cancer).*

*“At times I feel very isolated, it’s affecting my mood. I miss my friends” (female, breast cancer).*

*“My routine for physical activity has been completely changed, which has been very challenging to my motivation. But more than that it’s the social aspect and support that I really miss” (male, kidney cancer).*

*“I am more unsettled, nervous. I felt closed in and frighten at times. I believe Covid-19 has affected my mental health” (female, breast cancer).*

*“The restrictions have made an impact socially; the MM class was a great way of keeping each other motivated and encouraged. This is now not happening and does have a mental effect” (female, endometrial cancer).*

*“Feeling more anxious shopping. Groundhog Day feelings. Disturbed sleep & vivid dreams” (male, prostate cancer).*

*Physical impact*

*“My mobility has got worse, my health has deteriorated, and I have had a hospital stay for 9-days” (female, leukaemia).*

*“I have been unable to lose weight which I planned to do. I also feel as though my body is not as strong. There has been an overall change in my physical and mental health” (female, lymphoma).*

*“Put on a stone in weight. Bad back and joint problems came back. Feel teary and depressed. I stay longer in bed and going to bed later at night. Consuming more junk food, alcohol - its awful” (female, breast cancer).*

*“My level of fitness has fallen” (male, oesophageal cancer).*

*“Not able to join the social group means I’m not as motivated, less physically activity, more likely to not exercise” (male, chronic lymphocytic leukaemia).*

*“Going from trying to set up a routine which was mentally and physically beneficial was setback greatly” (male, prostate cancer).*

*“Yes, because we don’t have a coach I'm not doing right exercises and now my body needs toned all over again” (female, ovarian cancer).*

*Returning to Move More services*

*“I don't have many concerns, as long as we are confident that all the sports equipment, we use to carry out our exercises has been thoroughly cleaned for use. The hall is large and social distancing can be achieved. All participants are in the same boat and I trust that they too will be as vigilant as myself, look after each other” (female, breast cancer).*

*“Yes [concerns], but I have complete faith in my co-ordinator to mitigate against any potential problems.” (male, kidney cancer).*

*“Yes [concerned], would be nervous about getting the virus” (male, kidney cancer).*

*“No [concern], feel confident that measures will be put in place, so we have a safe environment.” (male, prostate cancer).*

*“No [concern] because I am confident strict safety measures will be put in place by Macmillan and adhered to by coordinator and participants” (female, breast cancer).*

*“On one hand I’m so looking forward to returning on the other I am concerned about how social distancing etc will happen” (female, breast cancer).*

*“Some concerns with regards to circuit training which I love, and these concerns would relate to hygiene...really the use of weights & handling of other equipment” (female, breast cancer).*

*Evaluation of the Macmillan response to COVID-19 restrictions*

*“With the online classes being streamed and the quiz recently I believe they are doing a great job" (male, prostate cancer).*

*“Our coordinator has been excellent as she has organised weekly zoom exercise classes which are fabulous and has also had a 1-1 session with me, via zoom, to direct me to other online sources on YouTube” (female, ampullary cancer).*

*“Our coordinator has done a good job of providing a weekly class on Zoom where we are able to chat and then do our exercises. We can also access a range of exercises provided by various coordinators through YouTube” (female, breast cancer).*

*“Our exercise class on Zoom is great if I use my laptop, I can see about 5 other participants. And it's good that the coordinators are doing this for us” (female, ovarian cancer).*

*“Apart from my lack of motivation I feel that my coordinator has been very supportive in what Move More has been able to do and although I haven't been able to join in I do appreciate being kept informed and I am thankful for what exercises are being offered which means I can do these as and when I feel that I am able to” (female, breast cancer).*

*“Good, versatile engagement and encouragement and as always non-judgemental" (male, colorectal cancer).*

*“I think my instructor is doing a brilliant job” (male, prostate cancer).*

Quotations relating to greater assistance:

*“Outdoor activities in small groups” (male, prostate cancer).*

*“As the majority of my class are in their 50s/60s technology is not one of our strengths so if there was any way of making it easier to take part in online classes it would be appreciated” (female, breast cancer).*

*“More outside activities during restrictions e.g. park exercise etc” (female, breast cancer).*

*“It would possibly help if we had difficulty with the technology ...that as older participants we would have someone to put our queries to if we were ' stuck ' at any point”(female, breast cancer).*

*Development of a MMNI App*

Positive:

*“I feel this would be very beneficial not only in the present situation but also at times when it is difficult to attend classes due to illness or travelling problems” (female, breast cancer).*

*“An app is good because you can exercise anytime, anywhere and also maybe do 2 or 3 types of exercise depending on what the app offers. It would be good for me when I return to work especially if Move More classes are on during the day. Participating in live supervised exercise classes are great too! People need encouragement and social interaction. I would need to know that I’m doing the exercises properly for a start. A mixture of both would be great!” (female, uterine cancer).*

*“It would be useful to have such an app” (male, prostate cancer).*

*“Would have no problem with using this form of technology” (male, colorectal cancer).*

Negative:

*“I would try it, but my technology skills are limited” (female, breast cancer).*

*“Possibly, but face to face interaction and social side is as important to participants as exercise itself” (male, chronic lymphocytic leukaemia).*

*“I would struggle to use an app, due to being of the older generation” (female, breast cancer).*

*“I like people close by and with technology friendships would be difficult to make. I think new participants couldn’t gel into the group and get the support they might require. I would miss the banter, the jokes and speaking with a number of people in the group” (male, prostate cancer).*
